# A Criticism of Hospital Reports

**Published:** 1909-05-08

**Authors:** 


### Resident Medical Officers' Department.

### A CRITICISM OF HOSPITAL REPORTS.

An "R.M.O." writes: This is the season of hospital reports, and many therefore to whom a well-printed page is a delight must just now be having their æsthetic sensibilities outraged, the only accompanying consolation being the small pleasure of detecting the mistakes of others and feeling consequent superiority. I allude to the grisly list of diseases which commonly figures in these documents, in one of which were found the following mistakes and blemishes: Eneurisis, tinuitis, alveola necrosis, dentigeious cyst, torti-Collis (torticollis not many lines below), Erbs' paralysis, sarcoma of sup. maxillary, Marion Simms for cervical carcinoma.

One could almost excuse an ill-educated and vulgar subscriber for exclaiming he would have "two each way on" the last! The reason for this yearly crop of errors is, of course, that the firms printing and publishing hospital reports have little experience in medical MSS. Yet I have no doubt that the services of a senior medical student as proof-reader could be got pretty easily.

[The first six in the above list are almost certainly misprints, and not mis-spellings. But with regard to the last two abbreviations, which are certainly both ungraceful and obscure, we would remind our correspondent that it may be with these as with the orthographical errors of the elder Dumas. When taxed with them he would reply airily that that was the printer's affair. Terms full of meaning on the case-sheet may become rather incomprehensible elsewhere. There is much to be said in favour of making the classification of diseases in a hospital report as general as possible. —Ed. *R.M.O. Department*, The Hospital.]

